# Factors Explaining Interpersonal Variation in Plasma Enterolactone Concentrations in Humans

**DOI:** 10.1002/mnfr.201801159

**Published:** 2019-03-26

**Authors:** Elin Hålldin, Anne Kirstine Eriksen, Carl Brunius, Andreia Bento da Silva, Maria Bronze, Kati Hanhineva, Anna‐Marja Aura, Rikard Landberg

**Affiliations:** ^1^ Department of Molecular Science Uppsala BioCenter Swedish University of Agricultural Sciences (SLU) Uppsala Sweden; ^2^ Diet, Genes and Environment Danish Cancer Society Research Center Strandboulevarden 49 DK 2100 Copenhagen Ø Denmark; ^3^ Department of Biology and Biological Engineering Food and Nutrition Science Chalmers University of Technology Gothenburg Sweden; ^4^ ITQB‐NOVA Instituto de Tecnologia Química e Biológica António Xavier Universidade Nova de Lisboa Oeiras Portugal; ^5^ Faculty of Pharmacy of the University of Lisbon Portugal; ^6^ Instituto de Biologia Experimental Tecnológica (iBET) Apartado 12 2781–901 Oeiras Portugal; ^7^ LC‐MS Metabolomics Center Kuopio Finland; ^8^ Department of Clinical Nutrition University of Eastern Finland Kuopio Finland; ^9^ VTT Technical Research Centre of Finland Ltd Post Office Box 1000, Tietotie 2 Espoo FI‐02044 VTT Finland

**Keywords:** determinants, enterolactone, interpersonal variation, lignans, plasma

## Abstract

Lignans are diphenolic plant compounds with potential health modulating properties that are absorbed to the circulation and metabolized to the enterolignans enterodiol (END) and enterolactone (ENL) by gut microbiota. Epidemiological studies have inconsistently shown that a high lignan intake and circulating ENL are associated with reduced risk of breast‐, prostate‐, and colorectal cancer as well as cardiovascular disease and total and cause‐specific mortality. Inconsistencies can be due to interpersonal variation of ENL formation or responses. The aim of this review is to identify and evaluate the impact of factors influencing variability in plasma concentrations of the main enterolignan, ENL. The main determinants of plasma ENL concentrations are intake of lignan and lignan‐rich foods, composition and activity of intestinal microflora, antimicrobial use, nutrient intake, BMI, smoking, sex, and age. Composition and activity of the intestinal microbiota appear to be the most critical factor governing interpersonal variability in plasma ENL concentration followed by the use of antibiotics. Future studies with combined data from gut microbiota and metabolomics with food intake and life style data can be used to estimate the relative contribution of the different factors to ENL concentration in quantitative terms.

## Graphical abstract

**Lignans are bioactive compounds from plants with potential health modulating properties**. Plant lignans are absorbed or passed through the colon and metabolized to the enterolignans enterodiol (END) and enterolactone (ENL) by gut microbiota. Studies on the role of lignans in human health, and ENL in particular, have been inconsistent. One reason for inconsistency can be due to large interpersonal variability in the factors that affect lignan concentrations and their activity in vivo. The current study describes the most important factors that determine the plasma ENL concentrations as well as the most important sources of interpersonal variability.

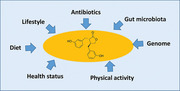

## 1 Introduction

**Figure 1:**
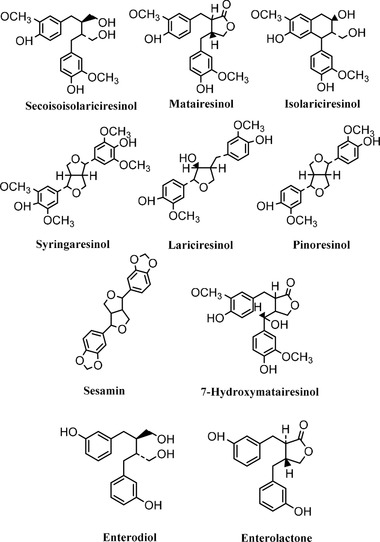
Chemical structures of common plant lignans and enterolignans. Adapted with permission.^50^ Copyright 2007, American Society for Nutrition. Adapted with permission.^72^ Copyright 2001, American Chemical Society.

Lignans are naturally occurring bioactive diphenolic plant compounds with potentially favorable effects on human health.^1^ Lignans are widely distributed in the plant kingdom and the most common plant lignans in the human diet include secoisolariciresinol (SECO), its glycosylated form secoisolariciresinol diglycoside (SDG), matairesinol (MAT), pinoresinol (PIN), lariciresinol (LAR), syringaresinol (SYR), sesamin (SES), 7‐hydroxymatairesinol (HMR), and isolariciresinol (isoLAR) (**Figure** 1). Plant lignans are converted to the mammalian lignans enterodiol (END) and enterolactone (ENL) by the gut microbiota in colon and are thereafter absorbed into the circulation^2^ (Figure 1).

Lignans belong to the phytoestrogens and exhibit both estrogenic and anti‐estrogenic activities in humans depending on the biological concentration of estradiol.^3^ Several years of intensive research have suggested, inconclusively, that high plasma concentrations of enterolignans are associated with a decreased risk of several cancers such as such as breast‐, prostate‐, and colorectal cancer but also with cardiovascular diseases and type 2 diabetes.^3^, ^4^, ^5^, ^6^, ^7^ Moreover, studies have shown reduced mortality rate in breast cancer patients among women with high plant lignin intake.^8^, ^9^, ^10^, ^11^ Lignans may mediate effects on disease risk through their phytoestrogen properties. They bind with highest affinity to estrogen receptor beta^12^ but the affinities for the estrogen receptor are ≈1000–10 000 times lower for lignans than for estradiol.^13^ They may also interact with activity of sex hormones through binding to sex‐hormone binding globulins, which results in higher free sex‐hormone levels.^14^ Moreover, enterolignans have modulating effects on angiogenesis and growth factors, indicating that ENL may affect breast carcinogenesis.^15^, ^16^ Animal studies support that the same factors may be of importance for prevention of early stage cancer leading to lower incidence as well as inhibition of the progression of already established tumors.^17^, ^18^ Among the lignans, ENL has been most widely studied in relation to different health outcomes. ENL has been associated with healthy lifestyle including no smoking, lower BMI, low alcohol intake, and higher intake of plant foods, and it has been suggested that ENL may be a biomarker of a healthy lifestyle rather than affecting cancer per se.^19^ Large interpersonal variation in the ENL concentration due to the above mentioned factors may be one reason behind the inconsistency of the reported health effects associated with high lignan intake.

The aim of this paper was to compile current information about the factors that influence total and interpersonal variation in plasma enterolignan concentration, with main focus on ENL. This information is important for the interpretation of the role of circulating ENL in chronic disease and for the interpretation of dietary intervention studies with lignan‐rich foods.

## 2 Literature Search Strategy

**Figure 2:**
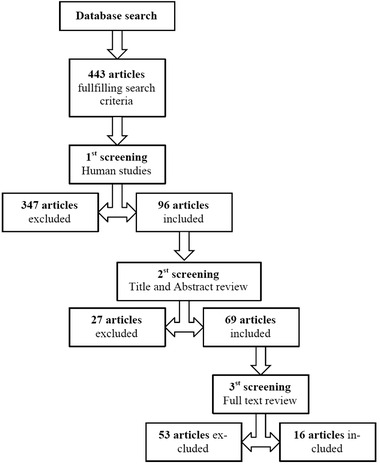
A flow diagram illustrating the retrieval process from the electronic databases PUBMED and WEB of SCIENCE.

A literature search was conducted according to scheme shown in **Figure** 2, by The Lignan working group within in the COST‐action network POSITIVe, using the following search terms and key words: HUMAN* AND (Lignan* OR Secoisolariciresinol* OR Matairesinol* OR Lariciresinol* OR Pinoresinol* OR Syringaresinol* OR Isolariciresinol* OR Arctigenin* OR Trachelogenin* OR Medioresinol* OR 1‐Acetoxypinoresinol* OR Secoisolar‐iciresinol di‐O‐glucoside* OR Sesamin* OR Sesamolin* OR Sesamolinol* OR Sesaminol* OR Sesaminol 2′‐O‐b‐D‐glucosyl (1→2)‐O‐[b‐D‐glucosyl (1→6)]‐b‐D‐glucoside* OR Sesaminol 2′‐O‐b‐D‐glucosyl (1→6)‐O‐b‐D‐glucoside* OR Ses‐aminol 2′‐O‐b‐D‐glucoside* OR Sesamol* OR Sesamolinol 4′‐O‐b‐D‐glucosyl (1→6)‐O‐b‐D‐glucoside* OR 7‐Hydroxymatairesinol* OR Isohydroxymatairesinol* OR Secoisolariciresinol‐sesquilignan* OR Cyclolariciresinol* OR 7‐Oxomatairesinol* OR Todolactol A* OR Conidendrin* OR Hydroxysecoisolar‐iciresinol* OR Nortrachelogenin* OR Lariciresinol‐sesquilignan* OR Anhydro‐secoisolariciresinol* OR Dimethylmatairesinol* OR Episesamin* OR Episesami‐nol* OR Sesaminol 2′‐O‐b‐D‐glucosyl (1→2)‐O‐b‐D‐glucoside* OR Enterodiol* OR Enterolactone* OR Sesaminol 2‐O‐triglucoside* OR Schisandrin* OR Gomisin D* OR Schisandrol B* OR Tigloylgomicin H* OR Schisanhenol* OR Schisantherin A* OR Gomisin M2* OR Deoxyschisandrin* OR Schisandrin B* OR Schisandrin C* OR 2‐Hydroxyenterodiol* OR 4‐Hydroxyenterodiol* OR 6‐Hydroxyenterodiol* OR 2‐Hydroxyenterolactone* OR 4‐Hydroxyenterolactone* OR 6‐Hydroxyenterolactone* OR 2′‐Hydroxyenterolactone* OR 4′‐Hydroxyenterolactone* OR 6′‐Hydroxyenterolactone* OR 5‐Hydroxyenterolactone* OR 7‐Hydroxyenterolactone) AND (Bioavailab* OR pharmacokinetic* OR kinetic* OR ADME OR identif* OR colon microb* OR colon microflora OR gut microb* OR urinary excretion OR biliary excretion OR enterohepatic* OR conjugat* OR Glucuronid* OR sulfat* OR sulphat* OR Mer‐captur* OR plasma OR urine OR interindividual varia* OR interpersonal varia* OR intraindividual varia* OR intrapersonal varia*) NOT drug‐interactions. Additionally, a document type search was included consisting of the key words (Article OR Book Chapter OR Correction OR Editorial Material OR Letter OR Note OR Proceedings Paper OR Review).

The total number of articles fulfilling the search criteria was 443. Of these studies, 96 were human studies that were included if they met the following criteria: 1) containing human data with information relevant to interindividual variation in absorption, distribution, metabolism, and excretion (ADME); 2) included main determinants of interindividual variation in lignan concentrations; and 3) contained information on proteins/genes involved in ADME of lignans. Relevant titles and abstracts were reviewed, resulting in 69 articles meeting the criteria, and hence included in the present analysis.

The 69 articles were screened in full text and included if relevant according to the criteria. Finally, 16 articles were selected and included in the study. A summary of the inclusion and exclusion of articles from the databases is shown in a flow diagram (Figure 2). Additional articles were retrieved from database links to and from original articles cited in the references. They were included based on the same criteria.

## 3 Factors Affecting Variation in Plasma Enterolactone

A wide range of plasma enterolacton concentrations has been found among individuals in epidemiological and experimental studies (**Table** 1). Factors affecting ENL concentration were identified and included intake of lignan‐rich foods, population characteristics such as sex, age, BMI, smoking habits and education level, and the composition and activity of intestinal microflora that depends on nutrient intake, health status, and use of antibiotics (**Table** 2). These factors are discussed in detail below.

**Table 1:** Pharmacokinetic parameters of plasma ENL after single/multiple dose/es of lignans or lignan‐rich foods

Subjects	*N*	Lignan intake	Single (S), multiple dose (M), or FQ (FQ)	Plasma ENL			References
				*C*_fasting_ (nmol L^–1^)	*C*_max_ (nmol L^–1^)	*T*_max_	
Women	1493	Traditional Finnish diet	FQ: 30 µg MAT/day 121 µg SECO/day	n/d	20.7 ± 0.6 (mean)	n/d	103
Women, post‐menopausal	23	Linseed	M: 25 g linseed/day; 2 weeks	11–15	140–818	n/d	104
Women, pre‐menopausal	9	Flaxseed	M: 25 g raw flaxseed per day; 8 days	6.90 ± 5.65 (SEM)	38.24 ± 20.04 (SEM)	8 days	79
Women, breast cancer‐free	728	Regular diet	FQ	n/d	8.4–26.1 (25th–75th percentile)	n/d	105
Women, breast cancer cases	365	Regular diet	FQ	n/d	8.0–25.1 (25th–75th percentile)	n/d	105
Women, breast cancer cases	194	Regular diet	FQ	n/d	19.6 ± 17.0 (mean)	n/d	106
Women, breast cancer‐free	208	Regular diet	FQ	n/d	25.9 ± 21.9 (mean)	n/d	106
Nonusers of oral antimicrobials	1789	Traditional Finnish diet	FQ	n/d	19.3 ± 16.1 (mean)	n/d	94
Users of oral antimicrobials	964	Traditional Finnish diet	FQ	n/d	16.4 ± 14.3 (mean)	n/d	94
Men	1359	Traditional Finnish diet	FQ: 46 µg MAT/day 126 µg SECO/day	n/d	17.8 ± 0.5 (mean)	n/d	103
Women (355) Men (288)	643	Regular diet	FQ: 0.963–1.016 mg lignansper day	n/d	10.2–12.5 (geometric mean)	n/d	62
Subjects	4	Sesame seeds	S: 50 g sesame seeds	n/d	<1.55–13.7	n/d	107
Women (6) Men (6)	12	Whole flaxseed	M: 0.3 g flaxseed/(kg body weight.day); 10 days	9.5 ± 1.1	29–262	n/d	108
Women (6) Men (6)	12	Crushed flaxseed	M: 0.3 g flaxseed/(kg body weight.day); 10 days	9.5 ± 1.1	22–277	n/d	108
Women (6) Men (6)	12	Ground flaxseed	M: 0.3 g flaxseed/(kg body weight.day); 10 days	9.5 ± 1.1	122–539	n/d	108
Women (3) Man (1)	4	Sesame seeds	S: 50 g sesame seeds (186.5 mg lignans)	0.60–9.90	65.4–1460	10–24 h	107
Women (5) Men (2)	7	Strawberry‐meal	S: 500 g strawberries	1.7–22.4	4–50	8–24 h	109
Subjects, adenoma cases	532	Regular diet	FQ	n/d	4.4–25.4 (25th–75th percentile)	n/d	91
Subjects, adenoma‐free	503	Regular diet	FQ	n/d	4.6–26.3 (25th–75th percentile)	n/d	91

**FQ**, food questionnaire; ***C*_fast_**, fasting concentration; ***C*_max_**, maximum concentration; ***T*_max_**, time of the maximum plasma concentration; **n/d**, not determined.

**Table 2:** The main factors influencing interpersonal variability in plasma ENL concentration

Factors	References
lignan‐rich food	20, 21, 22, 26, 28, 34, 39, 79, 87, 104, 110
Lignan intake	1, 24, 28, 29, 30, 31, 33, 36, 37, 38, 41, 42, 43, 62, 111
Sex	32, 34, 47, 48, 49, 50
Age	32, 48, 49
BMI	1, 32, 48, 49, 51, 52, 53
Smoking habits	32, 37, 52
Intestinal microflora	1, 47, 48, 50, 51, 63, 68, 69, 70, 73, 76, 77, 78, 79, 81, 82, 83, 84, 85, 86, 87, 89, 90
Nutrients intake	1, 48, 49, 65
Healthy status	1, 29, 98, 99, 100, 112
Antimicrobials	48, 91, 92, 93, 94, 95, 97

### 3.1 Lignans in Foods

Lignans are present in several, predominantly plant‐based foods.^20^, ^21^ Flaxseed (linseed) is the richest source of mammalian lignan precursors^20^, ^22^ as it contains two to three orders of magnitude more lignans than cereal grains, legumes, fruits, and vegetables. The major lignan is secoisolaricerecinoldiglucoside (SDG),^22^ along with lower content of matairesinol (MAT), pinoresinol (PIN), laricirecinol (LAR), and isolariciresicnol (isoLAR).^20^, ^22^ SDG is present in a complex‐bound form in the outer layers of the seed and ranges from 1.2–2.4 g/100g in defatted flour and 0.6–1.3 g/100 g in whole flaxseed flour 22. Lignan content in Sesame (*Sesamum indicum*) is comparable to flaxseeds^20^, ^21^, ^23^ and are the richest source of SES (≈60 mg/100 g) and PIN (≈30–50 mg/100 g).^20^, ^21^, ^24^ Mono‐, di‐, and triglucosides of sesaminol, sesamolinol, and PIN can be present in the oil free meal.^25^ Sunflower seeds (0.9 mg/100 g) and cashew nuts (0.6 mg/100 g) also have high lignan concentrations, especially of SECO and LAR, although isoLAR was also found in cashew nuts.^20^

Whole‐grain products, such as cereals (wheat, barley, and oats) and breads^22^, ^24^ are also important sources of lignans. Smeds et al. found the highest lignan content in rye and wheat, when compared with other cereals,^21^ with hydroxymatairesinol (HMR) as the dominant species, followed by syringaresinol (SYR) particularly in rye, where also SECO and MAT are found at high concentrations in the bran.

Legumes (bean, lentil, and soybean) and vegetables (broccoli, garlic, asparagus, and carrots) also contain relatively high levels of lignans, although only seldom in concentration above 1 mg/100 g.^20^, ^22^ Lower contents were found in dried apricots, dates, and prunes (<0.5 mg/100 g) as well as Yuzu (1.3 mg/100 g), a citrus fruit originating in East Asia.^20^ The lignans PIN and 1‐acetoxypinoresinol are typically found in olives and, consequently, in virgin olive oils.^26^

Lignans are also found in several beverages.^20^, ^24^ Among the nonalcoholic beverages, the highest lignan concentrations have been found in tea (40–80 µg/100 mL), followed by coffee (20–30 µg/100 mL), and juices such as orange and pomegranate juices (<10 µg/100 mL).^27^ Due to the presence of lignans in legumes, soya milk was found to contain 40 µg/100 mL.^27^ Several alcoholic beverages contain lignan such as red wine (80 µg/100 mL) with SECO and isoLAR as the most abundant ones, followed by beer (27 µg/100 mL; PIN and LAR) and white wine (22 µg/100 mL; LAR and SECO).^20^, ^24^

### 3.2 Lignan Intake

Plant lignan intake has been positively correlated with serum ENL concentrations.^1^, ^28^, ^29^ However, the food source and intake levels vary by population demographics, depending on habitual dietary patterns,^30^ variation in lignan content in commonly consumed foods,^31^ but also among individuals consuming the same diet over time.^32^ Other factors causing variability in reported lignan intake include differences in food composition databases, methodological differences,^21^ and number of mammalian lignan precursors included in the analysis.^33^

LAR and PIN contributed to 75% of average daily intake of lignans among a Dutch population and have accordingly been found to correlate more strongly to the total lignan intake than SEC and MAT.^34^ Also, total intake of SEC, MAT, LAR, and PIN was found to correlate more strongly with plasma ENL compared to SEC and MAT only.^35^ Consequently, epidemiological studies based on intake of four lignan precursors (SEC, MAT, PIN, and LAR) could therefore differ substantially in the classification of subjects, compared to studies only based on SEC and MAT.^34^

Various databases have been used to estimate the dietary intake of lignans in different populations, for example in Canada,^36^ Finland,^37^, ^38^ and Japan.^40^ These databases have different coverage of lignan compounds. In Finland, the main sources of lignans are seeds, cereals, fruit, berries, and vegetables,^37^ and the average intake of lignans was reported to 434 µg d^–1^,^37^ whereas Kilkkinen et al. (2003) estimated the mean lignan intake of Finnish men and women to only 173 and 151 µg d^–1^, respectively.^38^ In contrast, the average lignan intake by women in the United States has been estimated to 578 µg d^–1^, with the main source being fruit.^40^ Furthermore, the mean lignan intake by Dutch women was estimated to 560 µg d^–1^ and derived mainly from breads, nuts, and seeds primarily containing SEC.^41^ On the other hand, studies involving both SEC and MAT databases resulted in higher estimates of 1 mg d^–1^,^42^ and further increased to 1.24 mg d^–1^ when additional lignans were included^27^ in this particular population. Knowledge about the production of various lignan compounds in planta is still not complete and new mammalian lignan precursors are added continuously. For example, in 2007, rye was found to contain anhydro‐secoisolariciresinol, α‐conidendrin, todolactol A, and iso‐hydroxymatairesinol.^21^ The repertoire was further widened with the discovery of oligomeric lignans that were converted to monomeric units by microbiota in in vitro colonic model.^43^

This accentuates the fact that food consumption and databases are often not comparable between studies. More recent studies cover a wider array of lignans analyzed in foods, which may cause the somewhat higher estimations of total lignan intake than earlier studies.^44^ The variability of lignan intake estimates between studies, due to the above reasons, may complicate interpretation, especially in meta‐analyses.^33^

### 3.3 Sex

Women have been observed to have higher lignan content per calorie in their diet compared to men.^34^ In addition, constipation, which is associated with increased levels of ENL possibly due to increased lignan absorption from slower intestinal motility,^32^ is more frequent in women than men.^45^, ^46^ These factors may contribute to differences in serum ENL between men and women. Kilkkinen et al.^32^ investigated determinants of serum ENL concentration and found high variability between individuals, but with a higher ENL range in women (0–182.6 nmol L^–1^) compared to men (0–95.6 nmol L^–1^).^32^ As only a single sample per subject was examined, it is impossible to judge whether the large variation is due to within‐subject fluctuations and/or between‐subject differences or due to analytical measurement errors.^47^ However, the analytical variation is probably a minor contribution to the overall variation in comparison with fluctuations within and between individuals.^32^ Several studies have included adjustments for sex in statistical analysis of enterolignans in relation to health outcomes.^32^, ^48^, ^49^, ^50^

### 3.4 Age

Age appears to be an important factor related to circulating ENL levels. However, the underlying reason is not fully understood. Higher consumption of whole‐grain products and fruit and berries by older individuals combined with increased prevalence of constipation in the elderly^45^, ^46^ has been suggested to partly explain the positive association of age with serum ENL.^32^, ^34^, ^48^, ^49^

### 3.5 Body Mass Index

BMI has been inversely associated with plasma ENL in several studies.^1^, ^32^, ^49^, ^51^, ^52^ High BMI has, however, also been associated with lower intake of lignans,^34^ confounding potential underlying mechanisms. Kilkkinen et al.^32^ identified BMI as an independent predictor of serum ENL concentration in women but not in men. In the study, normal weight women had higher serum ENL concentrations compared to both underweight and obese women. It has been suggested that overweight and obese individuals overestimate their consumption of lignan‐rich food, since serum ENL concentrations were significantly lower compared to normal weight subjects even though their reported lignan intake were similar.^32^ However, the differences may also occur due to differences in gut microbiota composition and/or activity between obese and lean individuals as shown for other gut microbiota derived molecules.^53^

### 3.6 Race/Ethnicity

Race/ethnicity has been suggested as a factor influencing interpersonal variation in plasma ENL.^48^ Hernandez et al.^54^ and Cardet et al.^55^ included adjustments for race/ethnicity in the statistical analysis, comparing plasma^54^ and urinary^55^ phytoestrogen levels and dietary phytoestrogen intake. The biological rationale for a difference across race/ethnicity is yet unknown, but may include differences in diet, microbiota but also polymorphisms in genes involved in the metabolism of ENL.

### 3.7 Smoking

Smoking has been inversely associated with ENL concentration in several studies.^32^, ^38^, ^51^, ^52^ However, it should also be noted that smoking is associated with lower dietary intake of lignans^34^ and it is therefore difficult to judge to what extent smoking per se affects the ENL concentration in plasma. Smoking may also induce or inhibit metabolic enzymes in the formation or elimination of ENL.

### 3.8 Education Level

Education level has been suggested to influence interpersonal variation in plasma ENL,^48^ and a positive association has been observed between education and serum ENL concentration. However, after adjustment for other factors,^32^ the association disappeared indicating that healthy lifestyle (less smoking, lower BMI, and higher physical activity) among persons with a certain education level might be a confounding factor.^56^, ^57^

### 3.9 Nutrient Intake

Changes in dietary composition has been found to modulate the gut microbiome composition as demonstrated in several studies.^58^, ^59^, ^60^ For example, dietary fiber intake has been positively correlated with microbial diversity, thereby indirectly affecting the bioavailability of enterolignans.^61^ However, intake of dietary fiber and whole grain has also been directly associated with higher plasma ENL concentrations in several studies.^1^, ^34^, ^48^, ^49^, ^51^, ^52^, ^62^, ^63^, ^64^, ^65^ In a study by Horner et al.,^49^ dietary fiber accounted for 13% of the variability in plasma ENL concentration, among investigated determinants.

Fat intake has been negatively correlated with plasma ENL concentration,^51^ and suggested to induce an inhibitory effect on the microflora and diminishing diversity,^66^, ^67^ which may cause reduced synthesis and absorption of ENL. Horner et al., on the other hand, reported no significant association between fat‐related variables and plasma ENL. Further studies are needed to clarify the mechanistic role of high‐fat diet on microbial diversity and on ENL producing gut microbiota.

### 3.10 Intestinal Microbiota

**Figure 3:**
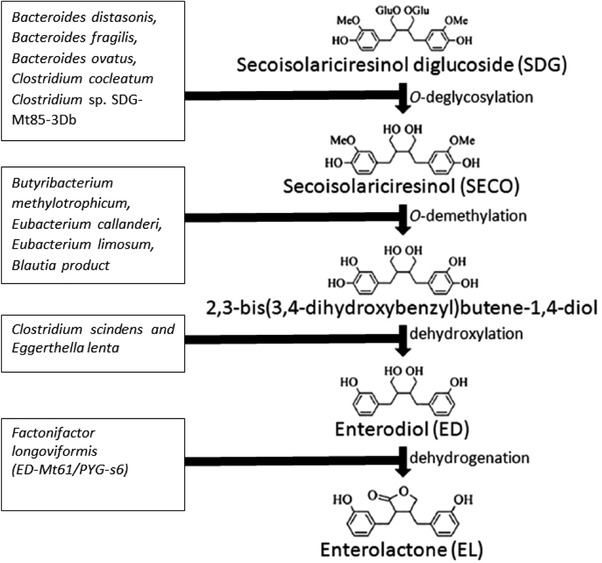
The main bacterial conversion steps of secoisolariciresinol diglucoside (SDG) to enterolactone (ENL) and examples of bacteria involved in the different steps. See text for references.

Interpersonal variation in plasma ENL has been linked to individual differences in lignan absorption and metabolism by intestinal microflora.^68^ Human intestinal bacteria are essential for the conversion of plant lignans to mammalian lignans,^2^, ^69^, ^70^ but no single bacterium can completely metabolize SDG to ENL.^71^ Instead, bacterial conversion, which involves deglycosylation, demethylation, dehydroxylation, and dehydrogenation^70^ (**Figure** 3), is catalyzed by a consortium of bacteria that share metabolic intermediates.^72^ Several bacteria have been identified to be involved in the different steps in the deglycosylation step, including strains of *Bacteroides distasonis*, *Bacteroides fragilis*, *Bacteroides ovatus*, *Clostridium cocleatum*, and *Clostridium* sp. SDG‐Mt85‐3Db, which was later named as *Clostridium saccharogumia* sp. nov.^73^ The demethylation step has been found to be catalyzed by strains of *Butyribacterium methylotrophicum*, *Eubacterium callanderi*, *Eubacterium limosum*, and *Blautia product* (former *Peptostreptococcus productus*).^74^ The dehydroxylation step is catalyzed by strains of *Clostridium scindens*, and *Eggerthella lenta*, whereas the dehydrogenation step is catalyzed by the strain ED‐Mt61/PYG‐s6,^71^ which was later named as *Lactonifactor longoviformis* gen. nov. sp. nov.^73^ Examples of bacterial species that have been associated with the conversion of SDG to ENL based on the literature mentioned above, are provided in Figure 3.

PIN is demethylated to SEC by *Egetherella lenta* and *Enterococcus faecalis*, Lar is similarly demethylated to SEC by *Egetherella lenta*.^75^ END is thereafter metabolized via ketone formation to ENL by *Lactonifactor longoviformis* gen. nov. sp. nov. MAT is already a ketone and its conversion to ENL requires only demethylation by the *Peptostreptococcus productus* 75 to 2,3‐bis (3,4‐dihydrobenzyl)butyrolactone, which is finally dehydroxylated to ENL. Borriello et al.^2^ demonstrated the importance of viable bacteria during conversion of END to ENL by human fecal flora, and that a bacterial concentration of up to 10^3^/g feces was required. Furthermore, depending on the intake of dietary precursors, several metabolic pathways operate to produce END and ENL.^2^

Lignan metabolism in the human gastrointestinal tract has also been found to be greatly influenced by specific bacteria with enantioselective dehydroxylation and oxidation capabilities.^76^ Clavel et al.^73^ observed that the strain ED‐Mt61/PYG‐s6 exhibit enantiospecific properties, thus only half of the initial concentration of END was converted to ENL in the study. Also, no ENL was detected when the strain was incubated with only (–)‐ED.^73^ In addition, Jin et al.^76^ observed enantioselective oxidation of END to ENL by the bacteria, *Ruminococcus* sp. END‐1 and END‐2. According to newer taxonomic classification, the strain END‐2 has been found to be more closely related to *Blautia producta* and *Blautia coccoides*.^77^ However, since other strains of *B. producta* are incapable of catalyzing dehydrogenation of END, it has been suggested that conversion of lignans by bacteria is strain‐specific.^78^ It remains to identify specific genes coding for enzymes that are involved in the pathway of converting lignans into enterolactone and to assess their presence across different microbial species.

The efficacy of converting different plant lignans into END and ENL varies between 0 and 100%, based on 24‐h in vitro studies.^72^ However, interpersonal variability in conversion rates seems to be very high,^79^ with strong indications that some individuals may lack the bacteria or appropriate intestinal environment necessary for oxidation of END to ENL.^79^, ^80^, ^81^ The cause of such variation as well as possible health effects have not been fully established.^79^, ^81^, ^82^, ^83^, ^84^, ^85^, ^86^

Already in 1982, Axelson et al.^69^ provided important insights into factors influencing the production of END and ENL, including microbiota composition, intestinal transit time, and the redox level of the large intestine. Clavel et al.^78^ discovered that the conversion of dietary lignans depends on the catalytic activity of both dominant and subdominant anaerobic bacterial communities in the human intestinal tract. In this study, ENL production was associated with *Peptostreptococcus productus* and *Clostridium coccoides* and with the presence of *Atopobium* group including *Eggerthella lenta*.^78^ Moreover, enterolignan‐producing microbial phenotypes have been associated with high diversity of microbial composition enriched in *Moryella* spp., *Acetanaerobacterium* spp., *Fastidiosipila* spp., and *Streptobacillus* spp.

Lagkouvardos et al.^87^ recently showed, in a small flaxseed intervention study, that overall diversity and composition of dominant fecal bacteria remained individual‐ specific during the study, and that *Ruminococcus bromii* and *Ruminococcus lactaris* were positively associated with ENL production.^87^

By analyzing the metabolism of SDG in human intestinal microbiota from one good and one moderate enterolignan producer, Eeckhaut et al.^88^ concluded that human intestinal microbiota is subject to large interpersonal variation,^89^ which is in accordance with another study.^90^ The cause of such variation warrants further investigations.

### 3.11 Antimicrobials

The use of oral antimicrobials is associated with decreased serum ENL concentration due to its major impact on the intestinal microbiota.^48^, ^91^ Earlier studies have suggested a required restoration period of 2 weeks for the intestinal microbiota to return to normal after use of antimicrobials.^88^, ^92^, ^93^ However, in more recent studies the effect of antimicrobial use on lowering serum ENL concentration persisted up to 12–16 months.^94^ In a recent Danish population‐based cohort study,^95^ the number of treatments and time since last treatment were both associated with serum ENL concentration, where more recent use of antimicrobials was associated with lower ENL concentrations, especially in women. Treatment with antimicrobials was associated with a 41% and 26% lower ENL concentration in plasma after use within <3 months and 3–12 months, respectively.^95^ In contrast, Horn‐Ross et al.^96^ studied the effect of antimicrobials on serum ENL, but found no significant difference between users and nonusers. However, lag‐time between usage and ENL measurement was unknown.

An inverse association between serum ENL and number of antimicrobial prescriptions has been observed, which is in accordance with earlier findings^97^ and provides further support for an important role of antimicrobials as a determinant of plasma ENL. Several possible effects of antibiotics on intestinal microbiota have been suggested, including interference with ENL formation from precursors and interference with enzymatic hydrolysis of ENL conjugates excreted in bile, reducing ENL reabsorption from the gut.^94^

Different antibiotics have various effects on serum ENL concentration, where macrolides have the strongest supressing effect with major impact in both the aerobic and anaerobic bacteria. On the other hand, amoxicillin, phenoxymethylpenicillin, and cephalosporins have been observed to cause only minor effects on intestinal microbiota. Moreover, interpersonal variation may influence the effect of antimicrobials on intestinal microbiota.^94^

### 3.12 Health Status

Postmenopausal and apparently healthy women with surgically removed breast cancer, and no detected metastasis, have been shown to have lower excretion of ENL in urine compared to postmenopausal healthy controls.^98^ In another case‐control study conducted among premenopausal women, women with breast cancer had significantly lower plasma ENL concentrations compared to their healthy controls.^29^ However, ENL concentration in plasma or urine was not associated with risk of developing breast cancer in the European Prospective Investigation into Cancer and Nutrition—Norfolk cohort,^99^ suggesting that it is disease status that affects ENL production rather than ENL concentrations that affect breast cancer development. Associations between plasma ENL concentrations and other disease states have been proposed, including lower risk of colorectal adenoma.^100^ In combination with specific genetic risk alleles for gastric cancer, enterolactone and other phytoestrogens showed interaction with gastric cancer risk. However, in this case, the hypothesis was that ENL and other phytoestrogens may block CagA, a major virulence factor of Helicobacter pylori, which is a major risk factor of gastric cancer. Disease status was not suggested to impact ENL levels.

The metabolic profile of postmenopausal women has been associated with lignan intake and enterolactone concentrations.^101^ Women in the highest quartile of enterolactone had lower BMI and fat mass, as well as better insulin sensitivity. They also consumed more fiber, which could have contributed to the observed effects on adiposity and insulin sensitivity.^101^ Whether these findings are due to differences in enterolactone concentrations or general lifestyle are unknown.

In general, it is difficult to draw conclusions about how enterolignan concentrations affects health status or vice versa. Most of the results are based on case‐control studies increasing the risk of reverse causation. As has been suggested, ENL may in fact be a marker of general lifestyle rather than being an active mediator in the disease etiology.

## 4 Final Remarks and Future Directions

The factors that affect plasma ENL concentrations and interpersonal variation in ENL include the intake of lignan and lignan‐rich foods, composition and activity of intestinal microflora, antimicrobial use, nutrient intake, BMI, smoking, sex, and age. Composition and activity of the intestinal microbiota seem to be the most critical factor governing interpersonal variability in plasma enterolignan concentration followed by the use of antibiotics, whereas the intake of lignan‐rich foods, constipation, and lifestyle factors such as smoking and BMI appear to explain only a small portion of the total variability between subjects in Western populations. Several plant‐lignans are poorly converted into ENL and it remains to elucidate whether they form other metabolites and to evaluate their potential effects on health. Moreover, the potential interactions between lignan‐derived metabolites such as ENL and other metabolites generated by gut microbiota from other precursor molecules from plant‐based foods such as phenolic acid metabolites, equol, *O*‐desmethylangolensin, resveratrol, urolithins remains to be investigated and could provide a basis for identification and stratification of subjects into different metabotypes. Untargted metabolomics approaches could be used to facilitate such investigations. Future studies could combine detailed data from gut microbiota with metabolomics, food intake, and health status data, to allow the relative importance of different factors in explaining plasma ENL variability to be estimated. For assessment of gut microbiota, metagenome analysis should be conducted to allow identification of bacterial species/strains with specific enzyme capacities of transforming plant lignans into ENL. Rapid and comprehensive high‐throughput targeted metabolomics should be used to assess the contents in foods for accurate intake assessment and to quantify the plasma and urine profiles of plant lignans.^102^ Studies are needed to characterize the pharmacokinetics and bioactivity of plant lignans as well as investigations to understand their potential impact on disease risk in prospective studies.

## Conflict of Interest

The authors declare no conflict of interest.
